# The Effects of Angiotensin Converting Enzyme Inhibitors (ACE-I) on Human N-Acetyl-Seryl-Aspartyl-Lysyl-Proline (Ac-SDKP) Levels: A Systematic Review and Meta-Analysis

**DOI:** 10.1371/journal.pone.0143338

**Published:** 2015-12-11

**Authors:** Ayanda Trevor Mnguni, Mark E. Engel, Megan S. Borkum, Bongani M. Mayosi

**Affiliations:** Department of Medicine, University of Cape Town and Groote Schuur Hospital, Cape Town, South Africa; Max-Delbrück Center for Molecular Medicine (MDC), GERMANY

## Abstract

**Background:**

Tuberculous pericardial effusion is a pro-fibrotic condition that is complicated by constrictive pericarditis in 4% to 8% of cases. N-acetyl-seryl-aspartyl-lysyl-proline (Ac-SDKP) is a ubiquitous tetrapeptide with anti-fibrotic properties that is low in tuberculous pericardial effusion, thus providing a potential mechanism for the heightened fibrotic state. Angiotensin-converting enzyme inhibitors (ACE-I), which increase Ac-SDKP levels with anti-fibrotic effects in animal models, are candidate drugs for preventing constrictive pericarditis if they can be shown to have similar effects on Ac-SDKP and fibrosis in human tissues.

**Objective:**

To systematically review the effects of ACE-Is on Ac-SDKP levels in human tissues.

**Methods:**

We searched five electronic databases (1996 to 2014) and conference abstracts with no language restrictions. Two reviewers independently selected studies, extracted data and assessed methodological quality. The protocol was registered in PROSPERO.

**Results:**

Four studies with a total of 206 participants met the inclusion criteria. Three studies (106 participants) assessed the change in plasma levels of Ac-SDKP following ACE-I administration in healthy humans. The administration of an ACE-I was associated with an increase in Ac-SDKP levels (mean difference (MD) 5.07 pmol/ml (95% confidence intervals (CI) 0.64 pmol/ml to 9.51 pmol/ml)). Two studies with 100 participants further assessed the change in Ac-SDKP level in humans with renal failure using ACE-I. The administration of an ACE-I was associated with a significant increase in Ac-SDKP levels (MD 8.94 pmol/ml; 95% CI 2.55 to 15.33; I^2^ = 44%).

**Conclusion:**

ACE-I increased Ac-SDKP levels in human plasma. These findings provide the rationale for testing the impact of ACE-I on Ac-SDKP levels and fibrosis in tuberculous pericarditis.

## Introduction

Tuberculous pericarditis is an important cause of heart failure in sub-Saharan Africa and other developing regions of the world where tuberculosis is endemic[1,2]. Constrictive pericarditis is a serious complication that occurs in 4–6% of cases of tuberculous pericarditis despite treatment with anti-tuberculous drugs and adjunctive corticosteroids[3]. Mutyaba and others investigated the causes of constrictive pericarditis, outcomes after pericardiectomy, and predictors of mortality in Cape Town, South Africa, during a 22-year period of high HIV/AIDS prevalence [4]. They found that TB was the main cause of constrictive pericarditis in South Africa, and that despite its efficacy at relieving the symptoms of heart failure, pericardiectomy was associated with high perioperative mortality of 16% that was not influenced by HIV status. New York Heart Association Functional Class IV and hyponatremia were predictors of early mortality after pericardiectomy [4]. TB pericarditis is associated with decreased levels of the anti-fibrotic tetrapeptide N-acetylseryl-aspartyl-lysyl-proline (Ac-SDKP) [5], whereas ACE-I’s are known to increase Ac-SDKP levels in rodent tissues [6]. Ac-SDKP is a potent anti-fibrotic agent and a negative regulator of hematopoietic stem cell differentiation. If ACE-I’s increase Ac-SDKP levels in human tissues, then they would be candidate drugs for use in TB pericarditis to prevent fibrosis and constriction[7,8] We conducted a systematic review of the literature to determine whether ACE-I’s increase Ac-SDKP levels in human tissues.

## Methods

The methods used were based on our protocol, which was registered in Prospero [9].

### Search Strategy

Two authors (ATM and MEE) undertook a systematic literature search of a number of databases for studies on the effects of ACE-I on human Ac-SDKP levels. Potentially relevant studies were selected on the basis of title and abstract for scrutiny without language restriction. The following databases where searched: PubMed, Google Scholar, EMBASE and the Cochrane Library. A combination of the following search terms (including the use of MeSH) was used: angiotensin-converting enzyme, angiotensin-converting enzyme inhibitors, human, *N*-acetyl-seryl-aspartyl-lysyl-proline, and Ac-SDKP. The search strategy is outlined in Table 1. The reference lists of identified articles were reviewed. Authors and experts undertaking research in the field of ACE-I and Ac-SDKP were also consulted. Studies selected for review were prospective observational studies of the effects of ACE-I on human Ac-SDKP levels.

**Table 1:** Pubmed search strategy (*adapted for use in other databases*).

#1	("angiotensin converting enzyme inhibitors" OR "ACE inhibitors")
#2	("N-acetyl-seryl-aspartyl-lysyl-proline level" OR Ac-SDKP level)
#3	(#1 AND #2) Filters: Humans

### Criteria for considering studies for this review

#### Types of studies

All prospective and observational studies were included.

#### Types of participants

Only studies with human participants were included.

#### Types of interventions

Interventions had to include any ACE-I, whether alone or as part of other interventions. Control intervention was any placebo.

#### Types of outcome measures

The primary outcome was the change in Ac-SDKP levels as detected by standardised laboratory assays/protocols following ACE-I administration in humans.

### Data Extraction and Management

Data were extracted by two authors (ATM and MEE) using a standardised data extraction form. Data were entered into Review Manager 5.1 statistical software for meta-analysis. Any disagreements on the eligibility of articles for inclusion were discussed with BMM.

### Quality Assessment

All articles included were critically appraised by two authors (ATM and MEE) for methodological quality in accordance with the methods of the Cochrane Collaboration[10]. Each article included was assessed for risk of bias based on sequence generation, allocation concealment, blinding, and incomplete outcome or missing data, where applicable. Heterogeneity between studies was assessed using the chi-square test set at a 10% level of significance[10]. The impact of any statistical heterogeneity was quantified using the I² statistic. If there was an acceptable degree of heterogeneity and it was appropriate to pool the data, the Mantel-Haenszel statistical method and Random Effects Analysis Model were used, with the results presented in the form of a meta-analysis.

### Data Synthesis and Analysis

Two authors (ATM and MSB) reviewed all the relevant articles identified from the search and, after scanning the titles, identified those that could potentially be included, subject to a reading of the abstracts. The full text of the articles was obtained for final evaluation for inclusion into the review according to the pre-specified inclusion criteria. The PRISMA guideline was used in reporting the findings of this review (S1 Text) [11]. The outcome (i.e. the effect of ACE-I on the Ac-SDKP level) was considered as a continuous variable. The outcome measure was calculated using risk ratios and 95% confidence intervals. Outcomes expressed in ng/ml were converted to pmol/ml by dividing the ng/ml value by the molecular weight of Ac-SDKP (487 Daltons) X 10^−3^. Interquartile ranges were converted to standard deviations as recommended in the Cochrane Handbook.[10]

## Results

**Fig 1:**
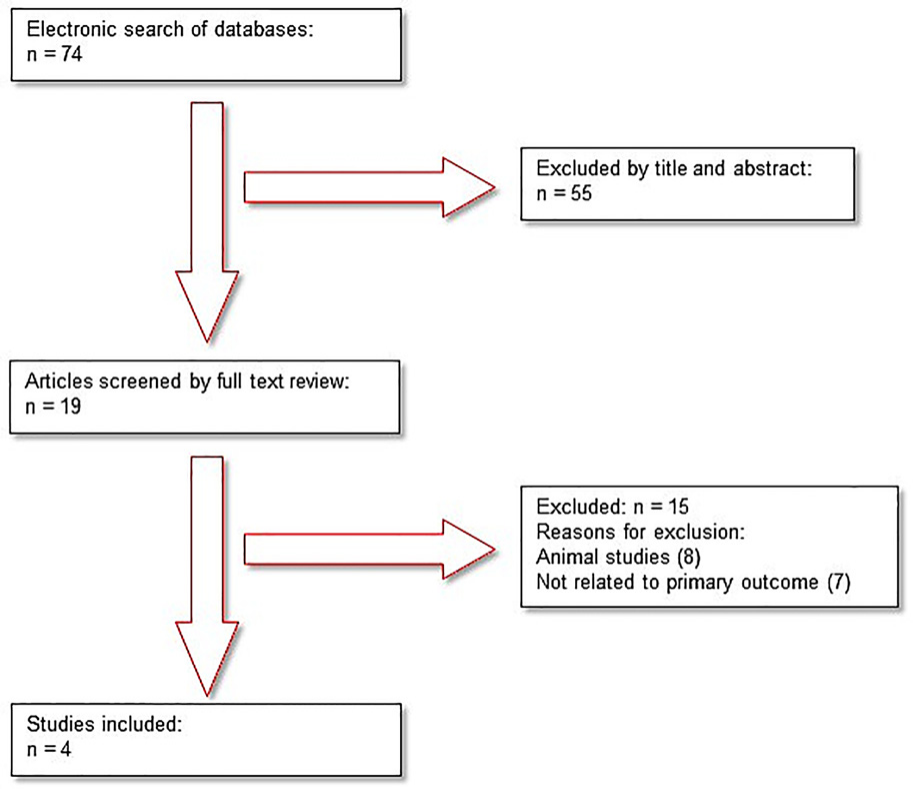
Flow diagram of search results.

Seventy-four papers where identified by the electronic search, of which 55 were excluded based on title and abstract (Fig 1). A further 15 papers were excluded following a full review of the text, as they were animal studies (n = 8) or not related to the primary outcome (n = 7). Thus, four studies met the inclusion criteria (Azizi 1996, Azizi 1997, Azizi 1999, Inoue 2010). The studies included were conducted in France (Azizi 1996, Azizi 1997, Azizi 1999) and in Japan (Inoue 2010). The studies in France included healthy subjects (Azizi 1996), patients with hypertension (Azizi 1997) and patients with renal failure (Azizi 1999). The study in Japan (Inoue 2010) included a combination of healthy patients and those with renal failure. The included studies are described in Table 2. The reasons for excluding studies that were initially considered relevant are provided in Table 3.

**Table 2:** Characteristic of studies included in the review.

Study ID	Methods	Participants	Intervention/Control	Outcome
**Azizi et al. 1996**[12]	Single-dose, double-blind two-way crossover study design set in Centre d’Investigations Cliniques, Hospital Broussais, Paris	16 healthy male Caucasian volunteers, age range 20 to 35 years	Intervention: captopril (50 mg) versus 50 ml of water (n = 8). Control = 8 patients who received placebo and 50 ml of water	Rise in Ac-SDKP level in blood following captopril administration
**Azizi et al. 1997**[13]	Prospective cohort study set at Broussais Hospital, Paris	50 white hypertensive patients of both sexes aged 18 to 75 years	Varying dosages of ACE-I were used;; 27 patients (21: M, 6: F) on ACE-I. Age range: 58+/-12 years, SBP: 164+/-33 mmHg; Control: 23 patients (17: M, 6: F) not on ACE-I. Age range: 55+/-8 years; SBP: 161+/-21 mmHg	Plasma Ac-SDKP levels elevated in patients on ACE-I
**Azizi et al. 1999**[14]	Observational study set at Broussais Clinical Investigation Centre	32 patients on the single oral dose; 12 patients on the multiple oral doses; 58 patients with CRF; 40 patients with normal renal function	Single oral dose study: 32 patients; Captopril (50 mg) with 25 ml of water; Multiple oral dose study: 12 patients; 10: Captopril (50 mg) with 25 ml of water; 2: placebo with 25 ml of water; 58 patients: 35 on ACE-I; 23 not on ACE-I; 40 patients with normal renal function: 19 on ACE-I; 21 not on ACE-I	Renal failure was associated with a slight increase in plasma Ac-SDKP levels; Ac-SDKP levels were increased in patients with normal renal function treated with an ACE-I,it was a moderate increase because ACE was intermittently reactivated between doses
**Inoue et al. 2010**[15]	Observational study set at Meiyo Clinic, Japan	41 patients: 7 healthy, 34 on dialysis; 28 dialysis patients: 10 on enalapril;; 18 on trandolapril	Existing patients on ACE-I–no dosages stated	Study focused on relatively simple and highly sensitive and specific analytical method for the quantitative determination of Ac-SDKP and Ac-SDKP minor in human plasma samples using SPE and LC-MS/MS in the MRM mode

ACE-I, angiotensin-converting enzyme inhibitor; CRF, chronic renal failure; SBP, systolic blood pressure; LC-MS/MS, liquid chromatography-tandem mass spectrometry; SPE, solid phase extraction; MRM, multiple reaction monitoring; Ac-SDKP minor, synthesised from thymosin β₁₀

**Table 3:** Characteristics of excluded studies.

Study ID	Reason for exclusion
Bogden et al., 1991[7]	Animal study
Cashman et al., 1994 [8]	Animal study
Comte et al., 1998[16]	Animal study
Struthers & MacFadyen, 1999[17]	Not related to primary outcome
Azizi et al., 2000 [18]	Not related to primary outcome
Azizi et al., 2001 [19]	Not related to primary outcome
Peng et al. 2003 [20]	Animal study
Cavasin et al., 2004[6]	Not related to primary outcome
Rasoul et al., n.d.[21]	Animal study
Azizi et al., 2006 [22]	Units of measurement provided as a ratio (Ac-SDKP/Creatinine)
Cavasin et al., 2007 [23]	Animal study
Lin et al., 2008[24]	Animal study
Liu et al., 2009[25]	Not related to primary outcome
Wang et al., 2010 [26]	Animal study
Nakagawa et al., 2012 [27]	Not related to primary outcome

### Change in Ac-SDKP levels in healthy participants

**Fig 2:**
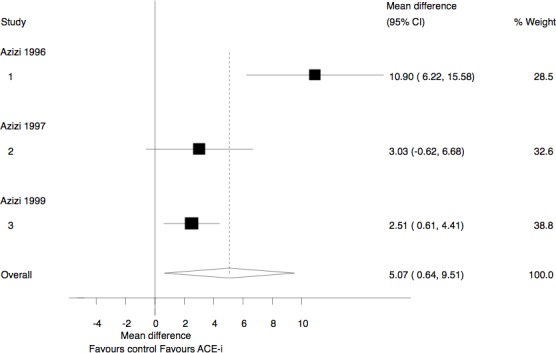
Change in Ac-SDKP levels in healthy participants. ACE-I, Angiotensin Converting Enzyme Inhibitors; IV, inverse variance.

Three studies (106 participants) assessed the change in the levels of Ac-SDKP following ACE-I administration in healthy humans [12,13,14]. Given the high statistical heterogeneity between studies (I^2 =^ 81%), a random-effects model was used. The administration of an ACE-I was associated with an increase in Ac-SDKP levels (mean difference (MD), 5.07 pmol/ml (95% confidence intervals (CI) 0.64 pmol/ml to 9.51 pmol/ml) (Fig 2). After exclusion of the trial with a small number of participants [12], the effect of ACE-I on Ac-SDKP levels remained significant, with a mean difference of 2.62 pmol/ml (95% CI 0.93 to 4.31).

### Change in Ac-SDKP levels in participants with renal failure

**Fig 3:**
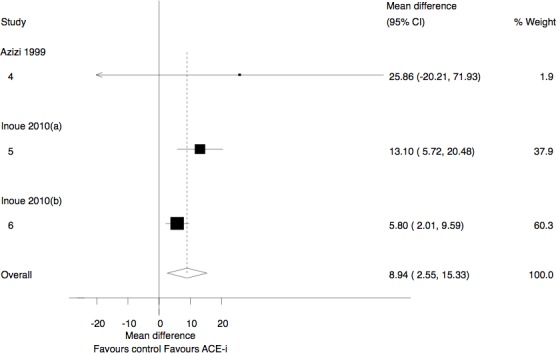
Change in Ac-SDKP levels in participants with renal failure. ACE-I, Angiotensin Converting Enzyme Inhibitors; IV, inverse variance.

Two studies with 100 participants assessed the change in Ac-SDKP level in humans with renal failure using ACE-I [15,28]. One study administered Captopril [28], while the second [15] used two types of ACE-I, namely enalapril (10 patients) and trandolapril (18 patients). The administration of an ACE-I was associated with a significant increase in Ac-SDKP levels (MD, 8.94 pmol/ml; 95% CI 2.55 to 15.33; I^2^ = 44%) (Fig 3). Unfortunately data was not available to allow for comparison with mean baseline levels within the ACE-I group.

### Methodological Quality

Table 4 shows the risk of bias assessment, which includes the components of random sequence generation, allocation concealment, blinding of participants and personnel, blinding of outcome assessment, incomplete data and selective outcome reporting. All these components were assessed as being either low risk, high risk or unclear. There were no missing data in any of the studies.

**Table 4:** Risk of bias assessment.

Study ID	Random sequence generation	Allocation concealment	Blinding of participants and personnel	Blinding of outcome assessment	Incomplete outcome data	Selective outcome reporting
Azizi et al. 1996[12]	Low risk	Unclear	Low risk	Low risk	Low risk	Low risk
Azizi et al. 1997[13]	N/A	High risk	High risk	High risk	Low risk	Low risk
Azizi et al. 1999[14]	N/A	High risk	High risk	High risk	Low risk	Unclear
Inoue et al. 2010[15]	N/A	High risk	High risk	High risk	Unclear	Unclear

N/A, not available.

## Discussion

This study has shown that ACE-I increases the plasma levels of Ac-SDKP in humans. This effect is present in health and disease, and appears to be a class effect of ACE-I. These findings are consistent with observations in animal models. However, no studies could be found on the impact of ACE-I in other body tissues such as the pericardium, nor were there studies on the effect of higher levels of Ac-SDKP on tissue fibrosis.

The hypothesis that treatment with ACE-I may increase the levels of Ac-SDKP in patients with TB pericarditis, which was put forward by Ntsekhe and others[5], is supported by this study. Our findings open the way for experiments to determine whether ACE-I can safely increase Ac-SDKP levels in pericardial fluid. However, the hypotensive effect of ACE-I may be deleterious in patients with haemodynamic instability caused by TB pericarditis. ACE consists of two catalytic domains–the C and N domains. The ‘C’ domain has a fivefold higher affinity for angiotensin 1, which is responsible for the maintenance of blood pressure control [29,30]. The ‘N’ domain is responsible for the degradation of the tetrapeptide Ac-SDKP. The ‘N’ domain not only plays an essential role in the degradation of Ac-SDKP but also plays a significant biological role, as is evident from a study that analysed bleomycin-induced lung injury in ACE C domain knockout (ACE^C^-KO) mice and ACE N domain knock out (ACE^N^-KO) mice. The ACE^N^-KO mice had significantly less bleomycin-induced lung fibrosis compared to ACE^C^-KO mice. This study confirmed that the inhibition of the ‘N’ domain of ACE was associated with significant endogenous anti-fibrosis signalling in the lungs [31]. Therefore, an ‘N’ domain catalytic-specific ACE-I, such as RXP407, may have great potential as an anti-fibrotic agent with minimal blood pressure effects in patients with haemodynamic instability such as tuberculous pericarditis. The affinity of ACE-I for the ACE catalytic domain is structure dependent. Zisman (1998)[32] was able to show that the hydrophobic moieties of ACE-I’s play an essential role in domain selectivity. Captopril was the first ACE-I used clinically, and it exhibited a threefold greater affinity for the ‘N’ domain than the ‘C’ domain. The newer ACE-I’s, namely enalaprilat, lisinopril and trandolapril, which have been developed for their antihypertensive properties, have been shown to display a higher affinity (approximately 24 times) for the ‘C’ domain [33]. This may explain the disparity in the change in Ac-SDKP levels across the studies using captopril compared to those using the newer ACE-I’s. The different dosages used across the studies may also explain the disparity in the change in AcSDKP levels across the various studies. One study assessed the analytical method best suited for the validation of AcSDKP. Mesmin et al used human urine and plasma to compare enzyme immuno assay and liquid chromatography/tandem mass spectrometry. He was able to show that tandem mass spectrometry, through the use of an internal standard, tailored sample preparation and chromatographic separation, had better intra- and inter-assay precision and allowed greater steadiness in intra-subject concentrations during the infusion.[34]

The inclusion of four small studies with a total of 206 participants from France and Japan may be seen as a limitation of this study. It is reassuring, however, that the direction of effect of ACE-I on Ac-SDKP was consistent and followed the biological expectation. The findings therefore have both internal and external validity and are likely to be of general relevance.

## Conclusion

ACE inhibition elevates Ac-SDKP levels in human plasma. These findings provide the rationale for the testing of the effect of ACE-I on Ac-SDKP levels in pericardial fluid with a view to reducing the incidence of constriction in tuberculous pericarditis.

## Supporting Information

S1 Text PRISMA Checklist. (DOC)

## References

[1] Sliwa K, Damasceno A, Mayosi BM. Epidemiology and etiology of cardiomyopathy in Africa. Circulation. 2005;112: 3577–83. 10.1161/CIRCULATIONAHA.105.542894 16330699

[2] Damasceno A, Sani M, Mondo C. The causes, treatment, and outcome of acute heart failure in 1006 Africans From 9 countries. Arch INTERN Med. 2012;172: 1386–1394. 22945249 10.1001/archinternmed.2012.3310

[3] Mayosi B, Ntsekhe M, Pandie S, Jung H, Gumedze F, Pogue J, et al Prednisolone and Mycobacterium indicus pranii in Tuberculous Pericarditis. N Engl J Med. 2014; 1–10. 10.1056/NEJMoa1407380 PMC4912834 25178809

[4] Mutyaba AK, Balkaran S, Cloete R, Badri M, Brink J, Sa FCS, et al Constrictive pericarditis requiring pericardiectomy at Groote Schuur Hospital, Cape Town, South Africa : Causes and perioperative outcomes in the HIV era (1990–2012). J Thorac Cardiovasc Surg. Elsevier Inc.; 2014; 10.1016/j.jtcvs.2014.07.065 25175954

[5] Ntsekhe M, Matthews K, Wolske J, Badri M, Wilkinson K a, Wilkinson RJ, et al Scientific letter: Ac-SDKP (N-acetyl-seryl-aspartyl-lysyl-proline) and Galectin-3 levels in tuberculous pericardial effusion: implications for pathogenesis and prevention of pericardial constriction. Heart. 2012;98: 1326–8. 10.1136/heartjnl-2012-302196 PMC4180888 22842991

[6] Cavasin M a, Rhaleb N-E, Yang X-P, Carretero O a. Prolyl oligopeptidase is involved in release of the antifibrotic peptide Ac-SDKP. Hypertension. 2004;43: 1140–5. 10.1161/01.HYP.0000126172.01673.84 15037553 PMC4677773

[7] Bogden a E, Carde P, de Paillette ED, Moreau JP, Tubiana M, Frindel E. Amelioration of chemotherapy-induced toxicity by cotreatment with AcSDKP, a tetrapeptide inhibitor of hematopoietic stem cell proliferation. Ann N Y Acad Sci. 1991;628: 126–39. Available: http://www.ncbi.nlm.nih.gov/pubmed/1648882 1648882 10.1111/j.1749-6632.1991.tb17230.x

[8] Cashman JD, Eaves a C, Eaves CJ. The tetrapeptide AcSDKP specifically blocks the cycling of primitive normal but not leukemic progenitors in long-term culture: evidence for an indirect mechanism. Blood. 1994;84: 1534–42. Available: http://www.ncbi.nlm.nih.gov/pubmed/8068944 8068944

[9] Mnguni T, Engel M. PROSPERO International prospective register of systematic reviews The effects of Angiotensin Converting Enzyme (ACE) -inhibitors on human N- acetylseryl-aspartyl-lysyl-proline (AcSDKP) levels : a protocol for a systematic review. 2014; 1–3.

[10] Higgins J, Green S. Cochrane Handbook for Systematic Reviews of Interventions. Higgins J, Green S, editors. The Cochrane Collaboration, 2009. Available: http://www.cochrane-handbook.org. London: Wiley-Blackwell; 2009 pp. 276–289.

[11] Moher D, Liberati A, Tetzlaff J AD. Preferred reporting items for systematic reviews and meta-analyses: the PRISMA Statement. Open Med. 2009;3: e123–30. 21603045 PMC3090117

[12] Azizi M, Rousseau A, Ezan E, Guyene T, Michelet S, Grognet J, et al Rapid Publication Acute Angiotensin-converting Enzyme Inhibition Increases the Plasma Level of the Natural Stem Cell Regulator N -Acetyl-Seryl-Aspartyl-Lysyl-Proline. J Clin Investig. 1996;97: 839–844. 8609242 10.1172/JCI118484 PMC507123

[13] Azizi M, Ezan E, Nicolet L, Grognet J-M, Menard J. High Plasma Level of N-Acetyl-Seryl-Aspartyl-Lysyl-Proline : A New Marker of Chronic Angiotensin-Converting Enzyme Inhibition. Hypertension. 1997;30: 1015–1019. 10.1161/01.HYP.30.5.1015 9369248

[14] Azizi M, Ezan E, Reny J-L, Wdzieczak-Bakala J, Gerineau V, Menard J. Renal and Metabolic Clearance of N-Acetyl-Seryl-Aspartyl-Lysyl-Proline (AcSDKP) During Angiotensin-Converting Enzyme Inhibition in Humans. Hypertension. 1999;33: 879–886. 10.1161/01.HYP.33.3.879 10082503

[15] Inoue K, Ikemura A, Tsuruta Y, Watanabe K, Tsutsumiuchi K, Hino T, et al Quantification of N-acetyl-seryl-aspartyl-lysyl-proline in hemodialysis patients administered angiotensin-converting enzyme inhibitors by stable isotope dilution liquid chromatography-tandem mass spectrometry. J Pharm Biomed Anal. Elsevier B.V.; 2010;54: 765–71. 10.1016/j.jpba.2010.10.009 21074346

[16] Comte L, Lorgeot V, Bignon J, Volkov L, Dupuis F, Wdzieczak-Bakala J, et al In vivo modifications of AcSDKP metabolism and haematopoiesis in mice treated with 5-fluorouracil and Goralatide. Eur J Clin Invest. 1998;28: 856–63. Available: http://www.ncbi.nlm.nih.gov/pubmed/9793000 9793000 10.1046/j.1365-2362.1998.00356.x

[17] Struthers M, MacFadyen R. Nonadherence with Angiotensin-Converting Enzyme Inhibitor Therapy. Am Coll Cardiol. 1999;34: 2072–7. 10.1016/s0735-1097(99)00439-8 10588226

[18] Azizi M, Massien C, Michaud a., Corvol P. In Vitro and In Vivo Inhibition of the 2 Active Sites of ACE by Omapatrilat, a Vasopeptidase Inhibitor. Hypertension. 2000;35: 1226–1231. 10.1161/01.HYP.35.6.1226 10856268

[19] Azizi M, Junot C, Ezan E, Ménard J, Cliniques I, Pompidou HG, et al ANGIOTENSIN I-CONVERTING ENZYME AND METABOLISM OF THE HAEMATOLOGICAL PEPTIDE N-ACETYL-SERYL-. Clin Exp Pharmacol Physiol. 2001;28: 1066–1069. 11903317 10.1046/j.1440-1681.2001.03560.x

[20] Peng H, Carretero O a, Brigstock DR, Oja-Tebbe N, Rhaleb N-E. Ac-SDKP reverses cardiac fibrosis in rats with renovascular hypertension. Hypertension. 2003;42: 1164–70. 10.1161/01.HYP.0000100423.24330.96 14581293 PMC6824434

[21] Rasoul S, Carretero OA, Peng H, Cavasin MA, Zhuo J, Sanchez-mendoza A, et al Antifibrotic effect of Ac-SDKP and angiotensin-converting enzyme inhibition in hypertension. J Hypertens. 2004;22: 593–603. 10.1097/01.hjh.0000098224.37783.55 15076166 PMC6824438

[22] Azizi M, Ménard J, Peyrard S, Lièvre M, Marre M, Chatellier G. Assessment of patients’ and physicians' compliance to an ACE inhibitor treatment based on urinary N-acetyl Ser-Asp-Lys-Pro determination in the Noninsulin-Dependent Diabetes, Hypertension, Microalbuminuria, Proteinuria, Cardiovascular Events, and Ramipril. Diabetes Care. 2006;29: 1331–6. 10.2337/dc06-0255 16732017

[23] Cavasin M a, Liao T-D, Yang X-P, Yang JJ, Carretero O a. Decreased endogenous levels of Ac-SDKP promote organ fibrosis. Hypertension. 2007;50: 130–6. 10.1161/HYPERTENSIONAHA.106.084103 17470726

[24] Lin C-X, Rhaleb N-E, Yang X-P, Liao T-D, D’Ambrosio M a, Carretero O a. Prevention of aortic fibrosis by N-acetyl-seryl-aspartyl-lysyl-proline in angiotensin II-induced hypertension. Am J Physiol Heart Circ Physiol. 2008;295: H1253–H1261. 10.1152/ajpheart.00481.2008 18641275 PMC2544498

[25] Liu X, Bellamy COC, Bailey M a, Mullins LJ, Dunbar DR, Kenyon CJ, et al Angiotensin-converting enzyme is a modifier of hypertensive end organ damage. J Biol Chem. 2009;284: 15564–72. 10.1074/jbc.M806584200 19307186 PMC2708853

[26] Wang M, Liu R, Jia X, Mu S, Xie R. N-acetyl-seryl-aspartyl-lysyl-proline attenuates renal inflammation and tubulointerstitial fibrosis in rats. Int J Mol Med. 2010;26: 795–801. 10.3892/ijmm 21042772

[27] Nakagawa P, Liu Y, Liao T-D, Chen X, González GE, Bobbitt KR, et al Treatment with N-acetyl-seryl-aspartyl-lysyl-proline prevents experimental autoimmune myocarditis in rats. Am J Physiol Heart Circ Physiol. 2012;303: H1114–27. 10.1152/ajpheart.00300.2011 22923621 PMC3517643

[28] Azizi M, Ezan E, Reny J-L, Wdzieczak-Bakala J, Gerineau V, Menard J. Renal and Metabolic Clearance of N-Acetyl-Seryl-Aspartyl-Lysyl-Proline (AcSDKP) During Angiotensin-Converting Enzyme Inhibition in Humans. Hypertension. 1999;33: 879–886. 10.1161/01.HYP.33.3.879 10082503

[29] Wei L, Alhenc-Gelas F, Corvol P, Clauser E. The two homologous domains of human angiotensin I-converting Enzyme are both catalytically active. J Biol Chem. 1991;266: 9002–9008. 1851160

[30] Rousseau A, Michaud A, Chauvet M, Lenfant M, Corvol P. The Hemoregulatory Peptide N-Acetyl-Ser-Asp-Lys-Pro Is a Natural and Specific Substrate of the N-terminal Active Site of Human Angiotensin-converting Enzyme. Biol Chem. 1995;270: 3656–3661. 10.1074/jbc.270.8.3656 7876104

[31] Li P, Xiao HD, Xu J, Ong FS, Kwon M, Roman J, et al Angiotensin-converting enzyme N-terminal inactivation alleviates bleomycin-induced lung injury. Am J Pathol. 2010;177: 1113–21. 10.2353/ajpath.2010.081127 20651228 PMC2928946

[32] Zisman LS. Inhibiting Tissue Angiotensin-Converting Enzyme. Circulation. 1998; 2788–2791. 9860776 10.1161/01.cir.98.25.2788

[33] Acharya KR, Sturrock ED, Riordan JF, Ehlers MRW. Ace revisited: a new target for structure-based drug design. Nat Rev Drug Discov. 2003;2: 891–902. 10.1038/nrd1227 14668810 PMC7097707

[34] Mesmin C, Cholet S, Blanchard A, Chambon Y, Azizi M, Ezan E. Mass spectrometric quantification of AcSDKP-NH2 in human plasma and urine and comparison with an immunoassay. Rapid Commun Mass Spectrom. 2012;26: 163–72. 10.1002/rcm.5326 22173804

